# Supplementary material for: Vitamin C Improves Oocyte In Vitro Maturation and Potentially Changes Embryo Quality in Cattle
Source: Vet Sci. 2024 Aug 13;11(8):372. doi: 10.3390/vetsci11080372 (PMC11360740; doi:10.3390/vetsci11080372)
Supplement: Supplementary file 1 [file vetsci-11-00372-s001.zip › vetsci-3068199-supplementary.pdf]

Table S1. Effects of VC on IVM of bovine oocytes

VC concentrations (μg/mL)	No. of oocytes	Maturation rate (%)
0	89	72.96 ± 1.02^{a}
25	78	75.52 ± 1.91^{a}
50	94	82.27 ± 0.65^{b}
100	81	73.98 ± 2.90^{a}

Note: The different lowercase in the same column shows significant difference（$p < 0.05$）. The same as in the following tables.

Table S2. Effects of VC on the development of bovine parthenogenetic embryos

VC concentrations(μg/mL)	No. of oocytes	cleavage rate (%)	blastocyst rate (%)
0	102	66.25 ± 1.84^{a}	14.79 ± 1.15^{a}
25	96	65.38 ± 4.10^{a}	13.79 ± 1.65^{a}
50	111	71.47 ± 0.76^{a}	15.01 ± 2.03^{a}
100	85	70.37 ± 4.08^{a}	15.46 ± 2.81^{a}

Table S3. Effects of VC on the development of bovine IVF embryos

VC concentrations(μg/mL)	No. of oocytes	cleavage rate (%)	blastocyst rate (%)
0	96	63.55 ± 1.14^{a}	11.99 ± 0.36^{a}
20	106	67.95 ± 0.95^{b}	12.94 ± 0.48^{a}
40	93	62.17 ± 1.42^{a}	11.59 ± 0.69^{a}
60	100	61.59 ± 0.92^{a}	11.32 ± 0.39^{a}

Table S4. Effects of VC combinations on the development of bovine IVF embryos

VC concentrations (μg/mL)	No. of oocytes	cleavage rate (%)	blastocyst rate (%)	No. of blastomeres
0/0	79	60.65 ± 1.20^{a}	11.79 ± 0.10^{a}	95.28 ± 2.86^{a}
50/0	88	64.42 ± 0.53^{b}	14.16 ± 0.80^{a}	93.60 ± 3.14^{a}
50/20	95	68.00 ± 1.53^{c}	14.55 ± 2.06^{a}	99.31 ± 2.80^{a}
